# lncRNA SLC16A1-AS1 as a novel prognostic biomarker in non-small cell lung cancer

**DOI:** 10.1136/jim-2019-001080

**Published:** 2019-07-31

**Authors:** Hong Yue Liu, Sheng Rong Lu, Zi Han Guo, Zhi Sheng Zhang, Xuan Ye, Qiong Du, Huan Li, Qiang Wu, Bo Yu, Qing Zhai, Jin Long Liu

**Affiliations:** 1 Department of Pharmacy, Fudan University Shanghai Cancer Center, Shanghai, China; 2 Department of Oncology, Shanghai Medical College, Fudan University, Shanghai, China; 3 Department of Pharmacy, The Central Hospital of Min-Hang District, Shanghai, China; 4 Department of Oncology, The Second Affiliated Hospital of Anhui Medical University, Anhui, China; 5 Department of Biotechnology and Pathology, School of Medical Technology, Shanghai University of Medicine & Health Sciences, Shanghai, China

**Keywords:** non-small cell lung cancer, SLC16A1-AS1, proliferation, prognosis

## Abstract

Long non-coding RNAs (lncRNAs) have proved to act as crucial biomarkers in tumors. Novel biomarkers in non-small cell lung cancer (NSCLC) need to be investigated badly. To identify the differentially expressed lncRNAs between NSCLC tissue and adjacent tissue, microarray analysis was performed. lncRNA SLC16A1-AS1 was significantly less expressed in NSCLC tissue than that in adjacent tissue. Gain-of-function experiments was performed to determine the biological functions of SLC16A1-AS. In situhybridization and survival analysis were applied in lung cancer tissue samples to determine the prognostic role of SLC16A1-AS1. It was showed that SLC16A1-AS1 was remarkably downregulated in NSCLC tissues and cell lines. Functionally, SLC16A1-AS1 overexpression could inhibit the viability and proliferation of lung cancer cell, block the cell cycle and promote cell apoptosis in vitro which may result from reduced phosphorylation of rat sarcoma (RAS)/ proto-oncogene serine/threonine-protein kinase (RAF)/ mitogen-activated protein kinase kinase (MEK)/ extracellular regulated protein kinases (ERK) pathway caused by elevated expression of SLC16A1-AS1. Clinical sample analysis showed that SLC16A1-AS1 had a favorable impact on the overall survival and progression-free survival of patients with NSCLC. Our results suggested that SLC16A1-AS1 may act as a potential biomarker for patients with NSCLC.

**Significance of this study**

**What is already known about this subject?**

- Long non-coding RNAs (lncRNAs) have proved to act as crucial biomarkers in tumors.
- Several lncRNA biomarkers in non-small cell lung cancer (NSCLC) have been reported.
- More novel lncRNA biomarkers need to be identified and investigated.

**What are the new findings?**

- SLC16A1-AS1 was remarkably downregulated in NSCLC tissues and cell lines.
- SLC16A1-AS1 overexpression could inhibit the viability and proliferation of lung cancer cell, block the cell cycle and promote cell apoptosis in vitro which may result from reduced phosphorylation of RAS/RAF/MEK/ERK pathway.
- SLC16A1-AS1 had a favorable impact on the overall survival and progression-free survival of patients with NSCLC.

**How might these results change the focus of research or clinical practice?**

- The results may provide new implication for novel therapeutic approaches for patients with NSCLC.

## Introduction

Lung cancer is a malignant tumor with the highest rate of morbidity and mortality worldwide. According to the International Agency for Research on Cancer,^1^ there were ~1.82 million new cases of lung cancer in 2012 throughout the world, which ranked first in incidence among all malignant tumors. The incidence of and mortality from lung cancer are very high.^2–4^ Non-small cell lung cancer (NSCLC) is the major type of lung cancer, accounting for ~80% of all cases of lung cancer.^5^ NSCLC is mainly divided into squamous cell carcinoma, adenocarcinoma, adenosquamous carcinoma, large-cell lung cancer, and sarcomatoid carcinoma. Owing to the lack of obvious clinical symptoms at an early stage, NSCLC is often detected at a late stage, with lymphatic and distant metastasis of tumor cells; therefore, the prognosis is poor, and the 5-year survival rate is usually <20%.^6^ NSCLC seriously threatens the human life and health. Therefore, exploring the underlying mechanisms of the occurrence and development of NSCLC is essential for the diagnosis and treatment of the disease. With the development of molecular biology technologies, long non-coding RNAs (lncRNAs) have gradually become a research hotspot, providing a new direction for tumor research. These molecules are named lncRNAs because the length of the transcribed RNA is >200 nucleotides and it does not encode a protein. lncRNAs play important roles in many biological activities, such as epigenetic, cell cycle, and cell differentiation regulation.^7^ Studies^8^ have found that lncRNAs are abnormally expressed in a variety of tumors, and lncRNAs with dysregulated expression can be tumor-promoting or tumor-suppressing factors. lncRNAs have multiple complex regulation mechanisms, such as encoding the upstream promoter of a protein-coding gene, interfering with the expression of downstream genes, mediating chromatin remodeling and histone modification, binding to transcription factors and specific proteins, and acting as a precursor for small RNAs.^9 10^ Research results have shown that lncRNAs can play an important role in the formation and progression of NSCLC by affecting a variety of signaling pathways. In this study, we screened for lncRNAs that are differentially expressed in NSCLC and paracancer tissues using a gene chip technology. We identified the functions of such lncRNAs and revealed their clinical significance, which provides new ideas on the pathogenesis of NSCLC and may guide clinical treatment.

## Methods

### Clinical samples

Lung cancer tissue and adjacent normal tissue that were wesected were obtained from Fudan University Shanghai Cancer Center from January 2004 to March 2017. Of them, seven NSCLC samples and five normal samples were selected for microarray profiling. Another 244 NSCLC samples with complete clinical information were used for immunohistochemical assay and survival analysis. Each participant was required for a signed informed consent. The Declaration of Helsinki was observed strictly.

### Microarray profiling

Differentially expressed lncRNAs were screened by the whole lncRNAs microarray expression profiling based on the criteria of fold change >2 and *Padj* <0.05. During experiment, we strictly followed the manufacturer’s standard protocols. Briefly, cyanine-3-CTP labeled cRNA was hybridized to lncRNA microarray chips. Samples were then analyzed on the chips after washing. R program was used to calculated and clustered the differentially expressed lncRNAs.

### Cell line culture

We purchased cell lines from American Type Culture Collection (Manassas, VA, USA) including NSCLC cell lines (A549 and NCI-H460) and human lung epithelial cells (BEAS-2B). Modified RPMI-1640 medium which was supplemented with 10% fetal bovine serum including 100 µg/L penicillin and 100 µg/L streptomycin was used to maintained cells involved in this study at 5% CO_2_ and 37°C.

### Real-time quantitative polymerase chain reaction

Total RNA was isolated by TRIzol reagent (Invitrogen, Carlsbad, CA, USA). Total RNA (1 µg) was reverse transcribed to cDNA by using Moloney Leukemia Virus Reverse Transcriptase Kit (Promega, Madison, WI, USA). SYBR Green Mix (Promega) was applied to amplify target primers. Sequences of the SLC16A1-AS1 primer is listed as follow: forward primer: TGGACGATGCATATGTGGGG; reverse primer: CACGTTGGTTATGCGGTCAC. All results were represented as 2^– ΔΔCt^. Endogenous control for SLC16A1-AS1 used gyceraldehyde 3-phosphate dehydrogenase (GAPDH).

### In situ hybridization

Cells were first trypsinzed and harvested. Poly-l-lysine-treated glass slides were used for seeding these cells. After about 24 hours, cells were fixed in methanol at −20°C for another 555 min. The assays were performed as previously described.^11^ Briefly, digoxigenin antibody (Roche, 11,093,274, 1:1000) was used to label a locked nucleic acid probe which contains complementarity sequences to a section of SLC16A1-AS1 (custom LNA detection probe, Exiqon). Then, the probe was synthesized. There were two independent pathologists to evaluate the intensity and the extent of staining. The pathologists were both blinded as a result of the experiment.

### Immunohistochemistry

Immunohistochemistry (IHC) staining was performed as previously described.^12^ Mouse monoclonal antibody against phospho-MEK1/2 (1:200) and phospho-RAF (1:200) were used in this assay. The pathologists were both blinded as a result of the experiment.

### Lentivirus production and cell transfection

The pBLLV-CMV-IRES-ZsGreen SLC16A1-AS1 cDNA lentiviral plasmid was purchased from Genelily BioTech Co. (Shanghai, China). The NSCLC cells which transfected with SLC16A1-AS1 cDNA lentiviral plasmid using Lipofectamine 3000 (Invitrogen) were selected by puromycin (2 µg/mL) for 2 weeks. Stable SLC16A1-AS1 overexpressed NSCLC cells were selected for further study. Transfection efficiency was verified by real-time quantitative polymerase chain reaction (RT-qPCR).

### Cell counting kit-8 assay

Cells (2×10^4^ cells/mL) were seeded and incubated on 96-well plates (100 µL/well) at 5% CO_2_ and 37°C for 24 hours. Each well was added by 10 µL cell counting kit-8 solution after 5 days culture. The cell proliferation was evaluated by absorbance values which is read by a microplate reader at a wavelength of 450 nm.

### Thiazolyl Blue Tetrazolium Bromide (MTT) assay

After incubation on 96-well plate (1×10^4^ cells/well) for 24, 48, 72 and 96 hours, each well was added by MTT (10 µL of 5 mg/mL) for incubation for another 4 hours. Then, removed the supernatants and added dimethyl sulfoxide (Thermo Fisher Scientific, Waltham, MA, USA) (100 µL/well). The cell viability was evaluated by absorbance values which is read by a microplate reader at a wavelength of 490 nm.

### Flow cytometry

The flow cytometry assays were performed as previously described.^13^ The Annexin V-FITC early apoptosis kit was used to determine cell apoptosis. The results were calculated by CellQuest software (BD Biosciences).

### Western blotting analysis

Western blotting analysis was conducted as previously described.^14^ Anti-RAF, anti-phospho-RAF, anti-MEK1/2 and anti-phospho-MEK1/2 were purchased from Sigma. Protein loading control used GAPDH.

### Statistical analysis

The statistical analyses were calculated by SPSS V.22.0 statistical software package and GraphPad Prism V.7.0. Numerical data were listed as mean±SD. The Student’s t-test or one-way analysis of variance was to compare the differences among groups. Overall survival (OS) was calculated from the time at diagnosis to the time of death of any cause. Progression-free survival (PFS) was calculated from the time that patients receive treatment to the time of disease progression. Log-rank test and Cox’s regression model were applied for univariate and multivariate analysis, respectively. P<0.05 was considered as statistically significant.

## Result

### lncRNA SLC16A1-AS1 was declined in NSCLC tissues and cell lines

**Figure 1:**
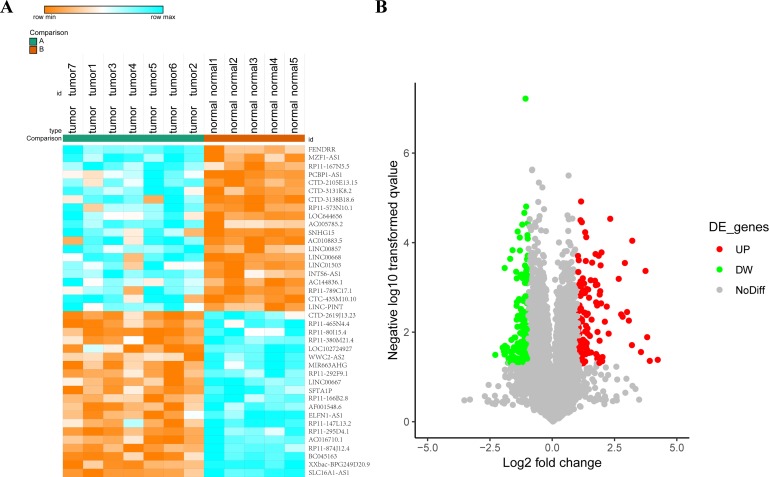
lncRNA SLC16A1-AS1 is downregulated in NSCLC tissues. (A) The top 40 differentially expressed lncRNAs (>1.5 fold; *Padj* **<**0.05) were showed in a heat map. (B) lncRNAs with fold change >1.5 and *Padj* **<**0.05 were colorfully plotted in the volcano plot. lncRNA, long non-coding RNA; NSCLC, non-small cell lung cancer.

There were 40 most differentially expressed lncRNAs (fold change >1.5, Padj <0.05) were listed (figure 1A, B). Among them SLC16A1-AS1 was significantly reduced in lung cancer tissues (figure 1A). lncRNA SLC16A1-AS1 was defined as it is an antisense transcript of gene SLC16A1. It localized in chromosome 1p13.2 with three exons. The biological characteristics of this lncRNA remained unclear.

### Upregulation of SLC16A1-AS1 inhibited cell proliferation

**Figure 2:**
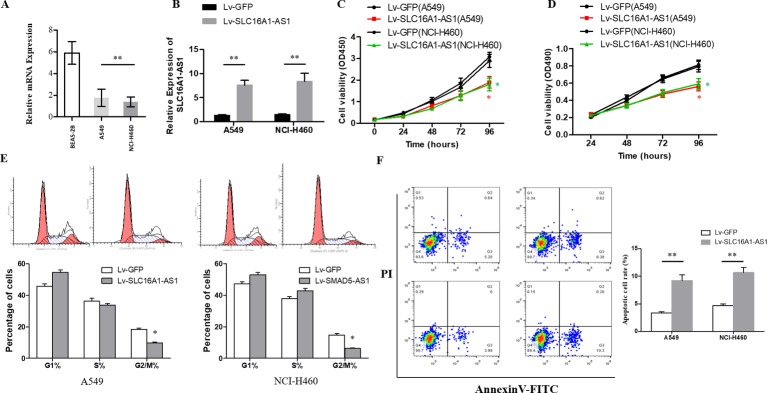
SLC16A1-AS1 inhibits cell proliferation of NSCLC cells in vitro. (A) The relative expression of SLC16A1-AS1 was measured by RT-qPCR in NSCLC cell lines (A549 and NCI-H460) and human lung epithelial cell (BEAS-2B). (B) Transfection efficiency was evaluated by RT-qPCR. ***P<0.001. Cell proliferation was evaluated by CCK8 (C) and MTT (D) assays. *P<0.01. (E) Flow cytometry system analysis was used to detect the phase of the cell cycle. *P<0.01. (F) Cell apoptosis rate was examined by Annexin V assay. **P<0.01. For each assay, three independent experiments were conducted. CCK-8, cell counting kit-8; NSCLC, non-small cell lung cancer;  RT-qPCR, real-time quantitative polymerase chain reaction.

The RT-qPCR results showed that the expression of SLC16A1-AS1 in NSCLC cell lines was significantly downregulated compared with human lung epithelial cells (BEAS-2B) (figure 2A), and difference was statistically significant (p<0.05). When transfected with SLC16A1-AS1 cDNA plasmid, the expression of SLC16A1-AS1 in A549 and NCI-H460 cells was obviously upregulated (figure 2B), the cell proliferation (figure 2C, D) and cell cycle (figure 2E) was significantly inhibited, and the cell apoptosis was promoted (figure 2F). These differences were statistically significant (p<0.05).

### Upregulation of SLC16A1-AS1 reduced the phosphorylation of RAS/RAF/MEK/ERK pathway

**Figure 3:**
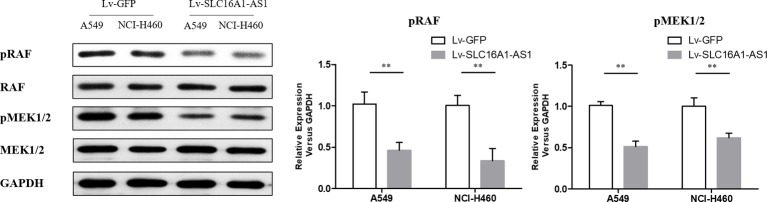
The phosphorylation levels of RAS/RAF/MEK/ERK pathway was detected by Western blotting analysis in A549 and NCI-H460 cells after transfection with control vector and Lv-SLC16A1-AS1. **P<0.01 versus control.

Western blotting analysis showed that the phosphorylation level of RAS/RAF/MEK/ERK pathway in A549 and NCI-H460 cells transfected with SLC16A1-AS1 cDNA plasmid was significantly inhibited compared with control group (figure 3). These differences were statistically significant (p<0.05).

### SLC16A1-AS1 had a favorable impact on the survival of patients with NSCLC

**Figure 4:**
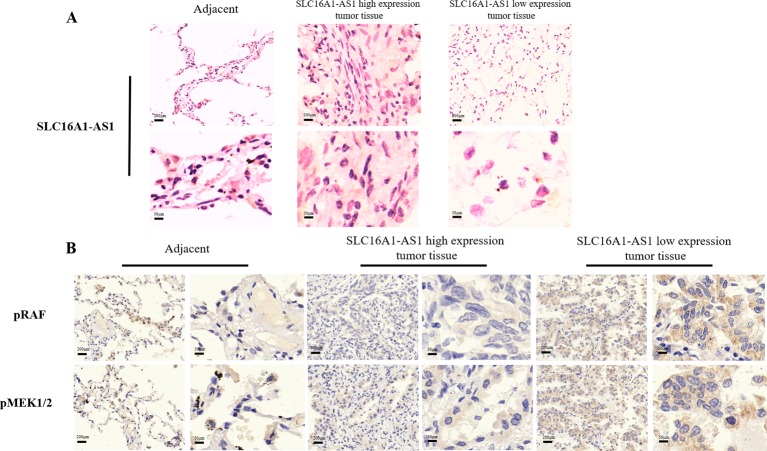
(A) In situ hybridization detection of SLC16A1-AS1 in NSCLC tissue (SLC16A1-AS1 high expression and SLC16A1-AS1 low expression) and adjacent normal tissue. (B) Immunohistochemistry detection of pRAF and pMEK1/2 in paraffin-embedded tissue sections.

**Figure 5:**
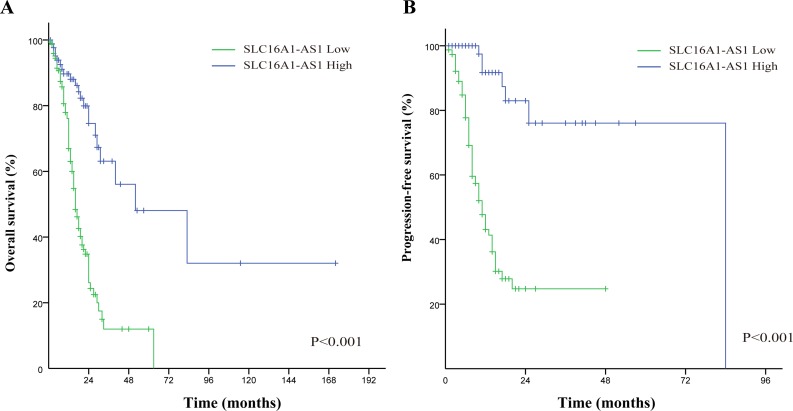
Kaplan-Meier survival curves: the overall survival (A) and progression free survival (B) in patients with NSCLC between low SLC16A1-AS1 group and high SLC16A1-AS1 group.

There were 244 NSCLC tissue with complete clinical information included in survival analysis. The results of in situ hybridization (ISH) manifested that the expression of SLC16A1-AS1 was lower in tumor tissues than that in normal adjacent tissues. SLC16A1-AS1 was also differentially expressed in tumor tissue (figure 4A). According to the SLC16A1-AS1 staining intensity in ISH, the intensity scored 0–2 was classified as low expression and the intensity scored 3–4 was classified as high expression. Compared with that in adjacent tissue and lung cancer tissue with high expression of SLC16A1-AS1, the phosphorylation of RAS/RAF/MEK/ERK pathway in lung cancer tissue with low expression of SLC16A1-AS1 was obviously promoted (figure 4B). The baseline clinical characteristics of all patients are listed in table 1. The median OS in high expression of SLC16A1-AS1 group was 52 months and it was 16 months in low expression of SLC16A1-AS1 group (figure 5A). The median PFS in high expression of SLC16A1-AS1 group was 84 months and it was 11 months in low expression of SLC16A1-AS1 group (figure 5B). This difference was statistically significant according to the univariate log-rank test (p<0.001). Multivariate analyses verified independent risk factors of OS included weight loss ≥5% (HR: 1.685, 95% CI 1.004 to 2.830, p=0.048); tumor, node, metastases (TNM) stage III–IV (HR: 2.274, 95% CI 1.174 to 4.405, p=0.015) and low expression of SLC16A1-AS1 (HR: 3.351, 95% CI 2.027 to 5.541, p<0.001) (table 2). Regarding of PFS, low expression of SLC16A1-AS1 also was identified as independent risk factor according to multivariate analyses (HR: 6.036, 95% CI 2.874 to 11.074, p<0.001) (table 3). These results manifested that SLC16A1-AS1 had a favorable impact on the OS and PFS of patients with NSCLC.

**Table 1:** Clinical characteristics of patients with non-small cell lung cancer

Characteristic	All patients	SLC16A1-AS1 (score 3–4)	SLC16A1-AS1 (score 0–2)	P value
Total	244	88	156	
Sex				**<0.001**
Male	156	41	115	
Female	88	47	41	
Age (years)				0.374
<70	188	65	123	
≥70	56	23	33	
Smoking				<0.001
No	147	84	63	
Yes	97	4	93	
BMI				**0.034**
<24	170	54	116	
≥24	74	34	40	
Pathological type				0.056
Adenocarcinoma	157	65	92	
Squamous carcinoma	72	20	52	
Others	15	3	12	
ECOG score				0.558
0–2	189	70	119	
3–4	55	18	37	
Weight loss				0.460
<5%	217	80	137	
≥5%	27	8	19	
TNM stage				0.512
I–II	42	17	25	
III–IV	202	71	131	
Vital status				**<0.001**
Deceased	102	21	81	
Alive	142	67	75	
Progression				**<0.001**
Yes	74	7	67	
No	170	81	89	

BMI, body mass index; ECOG, Eastern Cooperative Oncology Group; TNM, tumor, node, metastases; Bold font highlights a significant difference.

**Table 2:** Univariate and multivariate analysis of non-small cell lung cancer patients on overall survival

Variable	Univariate analysis		Multivariate analysis	
	HR (95% CI)	P value	HR (95% CI)	P value
Sex (male vs female)	1.357 (0.897 to 2.053)	0.149		
Age (≥70 vs <70 years)	1.095 (0.676 to 1.779)	0.711		
Smoking (yes vs no)	1.381 (0.918 to 2.078)	0.122		
BMI (≥24 vs <24)	0.543 (0.341 to 0.866)	**0.010**	0.789 (0.490 to 1.272)	0.331
Pathological type				
Adenocarcinoma	Reference			
Squamous carcinoma	1.209 (0.795 to 1.840)	0.374		
Others	0.930 (0.338 to 2.557)	0.888		
ECOG score (3–4 vs 0–2)	1.962 (1.286 to 2.995)	**0.002**	1.361 (0.875 to 2.117)	0.171
Weight loss (≥5% vs <5%)	2.296 (1.389 to 3.793)	**0.001**	1.685 (1.004 to 2.830)	**0.048**
TNM stage (III–IV vs I–II)	3.160 (1.664 to 6.002)	**<0.001**	2.274 (1.174 to 4.405)	**0.015**
SLC16A1-AS1 expression (low vs high)	3.858 (2.343 to 6.352)	**<0.001**	3.351 (2.027 to 5.541)	**<0.001**

BMI, body mass index; ECOG, Eastern Cooperative Oncology Group; TNM, tumor, node, metastases; Bold font highlights a significant difference.

**Table 3:** Univariate and multivariate analysis of non-small cell lung cancer patients on progression-free survival

Variable	Univariate analysis		Multivariate analysis	
	HR (95% CI)	P value	HR (95% CI)	P value
Sex (male vs female)	1.143 (0.704 to 1.854)	0.589		
Age (≥70 vs <70 years)	0.760 (0.417 to 1.385)	0.370		
Smoking (yes vs no)	0.913 (0.560 to 1.490)	0.716		
BMI (≥24 vs <24)	0.782 (0.467 to 1.308)	0.348		
Pathological type				
Adenocarcinoma	Reference			
Squamous carcinoma	1.343 (0.824 to 2.187)	0.237		
Others	1.338 (0.479 to 3.737)	0.579		
ECOG score (3–4 vs 0–2)	1.592 (0.950 to 2.669)	0.077		
Weight loss (≥5% vs <5%)	1.557 (0.796 to 3.043)	0.196		
TNM stage (III–IV vs I–II)	2.472 (1.213 to 5.036)	**0.013**	1.929 (0.948 to 3.927)	**0.015**
SLC16A1-AS1 expression (low vs high)	6.667 (3.150 to 12.518)	**<0.001**	6.036 (2.874 to 11.074)	**<0.001**

BMI, body mass index; ECOG, Eastern Cooperative Oncology Group; TNM, tumor, node, metastases; Bold font highlights a significant difference.

## Discussion

The abnormal expression of lncRNAs in lung cancer tissues is closely related to the occurrence and development of tumors. Su *et al* 15 studied the effect of lncRNA p53 regulation-associated lncRNA (PRAL) on lung cancer. The study found that the expression of PRAL in lung cancer tissues was significantly lower than that in paracancer and normal tissues, and the expression level of p53 was also significantly lower. Transfection of PRAL into the NCI-H929 and A549 cell lines promoted the transcription of p53 and inhibited the tumor cell proliferation. It has been reported that the expression of the plc00312 gene, located on the chromosome 3p25.3, was negatively correlated with the tumor size and was closely related to the development of NSCLC. Zhu *et al* 16 studied the role of linc00312 in NSCLC and found that the expression of linc00312 was downregulated in tumor tissues, which was consistent with the results of in vitro studies. The study also found a positive correlation between the expression of the transcription factor HOXA5 and linc00312, suggesting that linc00312 may play an important role in cell proliferation and tissue growth by activating the expression of the HOXA5 transcription factor. Urothelial carcinoma-associated 1 (UCA1) has been identified as an oncogene, and it is believed that dysregulated expression of UCA1 is closely related to tumorigenesis. The study^17^ found that UCA1 was highly expressed in NSCLC tissues and silencing of UCA1 could reduce the proliferation of tumor cells. Therefore, further studies of these abnormally expressed lncRNAs in NSCLC tissues may implicate them as potential biodiagnostic markers. Yang *et al* 18 found that lncRNA XLOC_008466 was highly expressed in NSCLC patients, and inhibition of the expression of XLOC_008466 inhibited the proliferation and invasion of tumor cells and promoted apoptosis. The study indicated that XLOC_008466 was similar in function to competing endogenous RNAs and could directly bind to miR-874 and downregulate its expression. Consequently, expression of MMP2 and XIAP, the downstream targets of miR-874, increased, suggesting that XLOC_008466 affects the cell proliferation and invasion via the miR-874-MMP2/XIAP pathway, thereby exerting a carcinogenic effect. lncRNA Gm15290 is highly expressed in NSCLC tissues. After Gm15290 was transfected into the NSCLC A549 cell line, the overexpressed Gm15290 could promote the cell proliferation and invasion. When Gm15290 was knocked down, proliferation and invasion of tumor cells were obviously reduced, and apoptosis was promoted. An RNA pull-down assay confirmed that Gm15290 could directly bind to miR-615–5 p, which inhibited the expression of miR-615–5 p, thus increasing the protein expression of miR-615–5 p target genes (insulin-like growth factor (IGF2), AKT serine/threonine kinase 2 (AKT2), and serine hydroxymethyltransferase 2 (SHMT2)), and promoted tumor cell proliferation. Metastasis-associated lung adenocarcinoma transcript 1 (MALAT1) can promote the tumor cell migration, invasion, and tumor growth and plays an important role in the development of NSCLC. Researchers used a small-molecule inhibitor of histone demethylase JMJD1A to bind to the promoter region of the MALAT1 gene to inhibit the expression of MALAT1, thereby inhibiting the tumor cell migration and invasion.^19 20^ It has been reported that inactivation of tumor suppressor genes plays an important role in tumorigenesis and tumor development. Chen Z *et al* 21 found that high expression of linc00473 was associated with frequent mutations and inactivation of the tumor suppressor gene *LKB1* in NSCLC. linc00473 can be used as a therapeutic target for NSCLC caused by *LKB1* inactivation. Insulin-like growth factor 2 antisense (IGF2AS) is considered an imprinted gene in Wilms tumors and is involved in the transcription and translation of various proteins.^22^ The study found that the expression of IGF2AS was low in NSCLC tissues and closely related to OS of patients. The results of in vitro functional experiments in lung cancer cell lines suggested that high expression of IGF2AS significantly inhibited the migration of tumor cells. The study also explored the signaling pathways associated with IGF2AS and found that the upregulated expression of IGF2AS inhibited the IGF2/vascular endothelial growth factor (VEGF)/basic fibroblast growth factor (bFGF) signaling pathway, thereby inhibiting the development of NSCLC, which suggests that IGF2AS is an ideal drug therapeutic target.^23^ HOX transcript antisense RNA (HOTAIR) is an lncRNA functioning as a trans-regulatory element, which negatively regulates the chromosome transcription and recombination and promotes the tumor progression. Studies have found that the bromodomain inhibitor I-BET151 could reduce the expression of HOTAIR via affecting its promoter, thereby playing a role in tumor suppression.^24^ In addition, Kruer *et al* 25 studied the mechanism of action of MEG3 and found that palbociclib treatment of A549 and SK-MES-1 lung cancer cells could activate the Rb pathway and increase the expression of MEG. Moreover, MEG3 affected the Rb pathway, thereby significantly reducing the proliferation of tumor cells. lncRNAs have broad application prospects in the treatment of NSCLC, but further research is needed for their application in clinical practice.

In this study, we found that the expression of lncRNA SLC16A1-AS1 was significantly lower in lung cancer tissues than in paracancer tissues, and this phenomenon was confirmed in cultured cells. The function of SLC16A1-AS1 in lung cancer is currently unclear. When the expression of SLC16A1-AS1 was exogenously increased in A549 and NCI-H460 lung cancer cells, the cell proliferation significantly decreased, the cell cycle was blocked, and the apoptosis rate increased. At the same time, we found that SLC16A1-AS1 regulated the proliferation of lung cancer cells by regulating the phosphorylation level of proteins in the RAS/RAF/MEK pathway. To further study the clinical application value of SLC16A1-AS1, we selected lung cancer tissue samples from 288 patients with a definitive diagnosis of NSCLC at our hospital for a retrospective study. All patients underwent regular treatment and regular follow-up at our hospital. The patients were divided into two groups according to the expression intensity score (0–4). Based on ISH, patients with 0–2 points were defined as the SLC16A1-AS1 low-expression group, and those with 3–4 points were defined as the SLC16A1-AS1 high-expression group. Multivariate analysis showed that SLC16A1-AS1 expression was an independent risk factor affecting OS and PFS of patients and was not affected by other factors, such as the TNM stage and Eastern Cooperative Oncology Group score. When OS and PFS of the two groups were compared, it was found that the median OS and PFS in the SLC16A1-AS1 high-expression group were significantly higher than those in the SLC16A1-AS1 low-expression group. These findings indicate that SLC16A1-AS1 may have a positive guiding value in the clinical treatment of NSCLC.

In summary, our results suggest that the expression of lncRNA SLC16A1-AS1 is reduced in NSCLC, and its biological function in NSCLC is antitumor proliferation, which was confirmed in in vitro experiments. Moreover, we discovered that the possible mechanism of action of lncRNA SLC16A1-AS1 is to regulate the phosphorylation level of proteins in the RAS/RAF/MEK pathway. A clinical retrospective study showed that lncRNA SLC16A1-AS1 had a clinical value in NSCLC and might be a new biomarker and a potential therapeutic target. To date, few studies have been published on lncRNA SLC16A1-AS1 worldwide. Its upstream and downstream regulatory mechanisms and in vivo effects are still unclear, and further research and exploration are needed. Meanwhile, prospective clinical studies with a large sample are required to further confirm its clinical value, and this will be the main goal of our next study.
